# Who gets enough sleep? – Sleep duration and classification based on sleep recommendations among the adult population in Germany

**DOI:** 10.25646/14574

**Published:** 2026-11-04

**Authors:** Marie Rissom, Natali Kaufholdová, Simon Löbl, Almut Richter, Kristin Manz, Julika Loss, Anja Schienkiewitz

**Affiliations:** 1 Robert Koch Institute, Department of Epidemiology and Health Monitoring, Berlin, Germany; 2 Institute for Medical Information Processing, Biometrics and Epidemiology – IBE, LMU Munich, Germany; 3 Pettenkofer School of Public Health, Munich, Germany

**Keywords:** Prevalence, Sleep duration, Sleep times, Weekend, Population groups, Telephone survey, Germany

## Abstract

**Background:**

An insufficient or excessive sleep duration can be associated with various health risks. The National Sleep Foundation recommends seven to nine hours’ sleep per night for 18 – 64-year-olds and seven to eight hours’ sleep for adults aged 65 and older. The aim of this study is to describe the sleep duration in the adult population in Germany and to identify population groups that meet the sleep duration recommendations.

**Method:**

Using data from the GEDA 2023 telephone survey, the mean sleep duration and the prevalence of adherence to sleep recommendations were calculated for weekdays, weekends and the whole week, stratified by gender, age group and educational group. In addition, bedtimes were grouped into categories and predicted probabilities of meeting the recommendations were estimated.

**Results:**

On average, the adult population in Germany sleeps for 07:26 hours per night, with longer sleep duration at the weekend, particularly among 18 – 29-year-olds (07:20 hours vs. 08:23 hours). On average, women sleep longer than men (07:31 hours vs. 07:20 hours). Overall, 51.6 % meet the recommended sleep duration, more frequently at weekends than on weekdays (54.7 % vs. 46.7 %) and among those with higher level of education (62.5 %). Later bedtimes are associated with a lower likelihood of meeting the recommendations.

**Conclusions:**

Continuous monitoring of sleep behaviour should be established as part of national health monitoring. Additional sleep parameters, such as sleep quality and sleep disorders, should also be taken into account to identify at-risk groups and to target public health measures effectively.

**Key messages**

► A sleep duration of seven to nine hours per night is recommended for 18 – 64-year-olds, and seven to eight hours for adults aged 65 and older; however, only 51.6 % of respondents meet these recommendations.
► Women are more likely to meet the recommended sleep duration than men.
► There are statistically significant differences in sleep duration between weekdays and weekends, particularly among young adults aged 18 to 29.
► On weekdays, going to bed early (at or before 22:00) is associated with a higher likelihood of meeting the recommended sleep duration.

**GEDA 2023**

German Health Update (data collection 2023)

**Data holder:** Robert Koch Institute

**Objectives:** Provision of reliable information on the health status, health behaviour and health care of the population living in Germany

**Study design:** Cross-sectional telephone survey

**Population:** German-speaking population aged 18 years and older living in private households that can be reached via landline or mobile phone

**Sampling:** Random sample of landline and mobile telephone numbers (dual-frame method) from the ADM sampling system (Arbeitskreis Deutscher Markt- und Sozialforschungsinstitute e.V.)

**Sample size:** Approximately 1,000 participants per questionnaire module per month

**Data collection period:** January 2023 – February 2024

**GEDA survey waves:** GEDA 2009, GEDA 2010, GEDA 2012, GEDA 2014/2015-EHIS, GEDA 2019/2020-EHIS, continuous survey between 2021 and 2023

Further information is available at www.rki.de/geda-en

## 1. Introduction 

Adequate sleep duration is a key determinant of health and can influence both physical and mental well-being. A persistently insufficient (less than seven hours) or excessive (more than nine hours) sleep duration is associated with a wide range of health risks [1]. Accordingly, the literature also refers to a U-shaped relationship between sleep duration and health outcomes, whereby sleeping for too little or too long can have negative effects on health [2, 3]. For instance, insufficient sleep can increase the risk of high blood pressure, obesity and diabetes, as well as neurological consequences such as memory loss [3–9]. Furthermore, a short sleep duration increases the risk of overall mortality [10]. Sleeping for too long is also associated with an increased risk of mortality (particularly when sleeping for more than ten hours). Women with irregular and prolonged sleep durations are especially affected [5, 7]. In addition, a long sleep duration may contribute to dementia or Alzheimer’s disease [3, 4], as well as weight gain or diabetes [3, 4, 6]. 

An individual’s sleep duration can be influenced by various internal and external factors. Internal factors include, for example, biological sleep preference (chronotype), age and gender, whilst external factors may relate to employment and working hours, or shift work [11–13]. Review studies suggest that, in addition to gender differences, sleep duration also varies between age and educational groups [14–16]. 

It is also important to distinguish between weekdays and days off, such as the weekend. Whilst for many people the time they get up on weekdays is largely dictated by external commitments, such as work or education, they often have greater flexibility over when they get up on days off [13, 17]. Consequently, bedtimes and wake-up times at the weekend can differ significantly from those on weekdays, which can also have an impact on the sleep duration [12, 13, 17]. In this context, so-called ‘social jet lag’ also plays a role: this refers to a deviation in sleep times that arises when societal time structures, such as working hours, do not align with individual biological sleep preferences. As a result, sleep times and sleep duration at the weekend may shift compared to weekdays, which can also have negative effects on health [18].

Several studies, particularly from an international context, have already examined the links between sleep duration and health outcomes. Accordingly, the National Sleep Foundation (NSF) has developed age-specific recommendations on sleep duration for children and adults through a panel of experts. For adults aged 18 – 64, a sleep duration of seven to nine hours per night, and for adults aged 65 and older, a sleep duration of seven to eight hours per night, is recommended [1]. The recommendations are designed to promote long-term physical and cognitive health throughout the lifespan and to reduce the risk of chronic diseases and mortality. A distinction must be made between the NSF’s recommendations and subjective sleep need, which describes the individually perceived amount of sleep a person requires. This can vary from person to person. The optimal sleep duration for each individual may also differ and depend on various factors. It is therefore difficult to define a single sleep duration that is equally valid for everyone [1, 19]. 

Although the NSF’s recommendations are based on US studies, they are also used in European countries to assess sleep behaviour in the absence of national guidelines [14, 20, 21]. Their application enables a comparable classification of sleep duration and contributes to the international comparability of results. 

In Germany, studies on adults’ sleep behaviour tend to focus on clinical aspects or very short observation periods, meaning that it is not possible to draw conclusions about seasonal effects or generalise to the population as a whole [22, 23]. The results of these studies show that a large proportion of the participants sleep for no more than seven hours [22, 23]. There is a lack of current, population-level data on compliance with healthy sleep duration recommendations in Germany, underlining the need for such data. 

The aim of this study is to describe the sleep duration of the adult population in Germany and to characterise population groups that meet the sleep duration recommendations.

## 2. Method

### 2.1 Sample design and study methodology

Data from the ‘German Health Update’ (GEDA), conducted by the Robert Koch Institute (RKI), were used for the present analyses (Infobox). GEDA is a representative cross-sectional survey of adults. Questions on sleep behaviour were asked between May 2023 and February 2024 as part of GEDA 2023 (n = 8,026). Data collection was carried out using a standardised, computer-assisted telephone interview (CATI). Participants were selected via a random sample of landline and mobile numbers (dual-frame method) [24]. A more detailed description of the study methodology can be found in Allen et al. [25]. 

A total of 677 individuals reported sleeping less than three hours or more than 13 hours on both weekdays and at weekends were excluded from the analyses [26, 27]. Thus, 7,349 individuals were included in the analyses. 

### 2.2 Variables

**Gender: **To describe gender differences, GEDA collected data on both the sex assigned at birth and gender identity (including an open response option). The analyses by gender include individuals who identify as female or male. Gender- diverse people who do not identify with these categories are not reported separately in the analyses by gender due to the small number of cases [28].

**Age groups: **The age of the participants was calculated based on the year and month of birth provided, as well as the date of the survey. The following four age groups were then defined: ’18 – 29 years’, ’30 – 44 years’, ’45 – 64 years’ and ‘65 years and older’.

**Educational groups: **The CASMIN classification (Comparative Analysis of Social Mobility in Industrial Nations) was used to define educational groups. For the analyses, three groups were defined: ‘low’ (primary or lower secondary education), ‘medium’ (intermediate or higher vocational education/training) and ‘high’ (higher education) [29]. 

**Effective sleep duration and bedtimes: **Participants were asked the following questions regarding their sleep behaviour on weekdays: ‘At what time did you usually go to bed in the evening on weekdays or working days over the last four weeks?’ and ‘At what time did you usually wake up in the morning on weekdays or working days?’ For weekends, the questions were asked in the same way: ‘At what time did you usually go to bed in the evening on weekends or non-working days over the last four weeks?’ and ‘At what time did you usually wake up in the morning on weekends or non-working days?’ 

To determine the actual effective sleep duration in the format hours:minutes, the difference between the time of waking and the time of going to bed was calculated, taking into account the time taken to fall asleep. 

For bedtimes, the following four categories were established for both weekdays and weekends: at or before 22:00, > 22:00 – 23:00, > 23:00 – 24:00 and after 24:00. For the analyses of adherence to sleep recommendations, a binary variable (Yes/No) was created indicating whether the respondent met the recommendations (18 – 64 years: seven to nine hours; 65 years and older: seven to eight hours) [1, 19]. 

### 2.3 Statistical methods

The mean sleep durations were calculated with 95 % confidence intervals. The means were calculated separately for weekdays, weekends and the whole week. Sleep duration for the whole week was calculated as follows: 



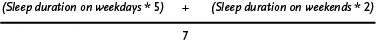



Differences between men and women were tested using t-tests, taking the sample design into account. For characteristics with more than two categories (age group, educational group), weighted linear regression models followed by a Wald test were calculated. A p-value < 0.05 was considered statistically significant.

Furthermore, weighted prevalences with 95 % confidence intervals were calculated for the defined sleep duration categories. The analyses were carried out for the whole week, as well as separately for weekdays and the weekend. All analyses were stratified by gender, age group and educational group. 

To determine which bedtime windows are associated with meeting the sleep duration recommendations, predicted probabilities from logistic regression models were calculated for the categories of bedtimes, separately for women and men and adjusted for age and educational groups. 

A weighting factor was applied to correct for variations within the sample with regard to gender, age, educational level and German federal state [25]. All analyses were carried out using R version 4.4.1 and RStudio 2026.01.02 (dataset version dated 16 April 2025), and survey procedures for complex samples were utilised [30].

## 3. Results 

### Description of the study population

Table 1 presents a description of the characteristics of the entire study population (n = 7,349; women n = 3,878, men n = 3,436). After weighting, the proportion of women was 51.5 % and the proportion of men was 48.5 %.

### Sleep duration

The average sleep duration in the general population is 07:26 hours (Table 2). On weekdays, adults sleep for a statistically significantly shorter duration than at the weekend (07:15 hours vs. 07:53 hours, p < 0.01). Women sleep statistically significantly longer than men (07:31 hours vs. 07:20 hours, p < 0.01). The shortest sleep duration is found among 45 – 64-year-olds (07:15 hours), particularly on weekdays (07:03 hours). Young adults aged 18 – 29 sleep over an hour longer at the weekend than on weekdays (08:23 hours vs. 07:20 hours). Differences between educational groups are small but statistically significant on weekdays and over the whole week. Adults in the medium educational group sleep the least on weekdays (07:12 hours), whilst there is hardly any difference between the low and high educational groups (07:18 hours vs. 07:20 hours). 

### Adherence to the recommended sleep duration

Overall, half of the respondents (51.6 %) meet the sleep recommendations (Figure 1). It is evident that the recommendations are met more frequently at the weekend (54.7 %) than on weekdays (46.7 %).

There are also gender-specific differences: 54.0 % of women and 49.1 % of men meet the recommended sleep duration over the whole week. The higher prevalence at weekends compared with weekdays is observed for both genders (women: 57.3 % vs. 49.0 %; men: 52.3 % vs. 44.5 %).

In terms of age groups, 18 – 29-year-olds (61.1 %) and 30 – 44-year-olds (59.4 %) are most likely to meet the recommendation. Clear differences between weekdays and weekends are also evident within the age groups. The 45 – 64 age group shows the greatest difference: At the weekend, 61.0 % of participants meet the sleep recommendations, compared with just 47.7 % on weekdays. The group aged 65 and older is the least likely to meet the sleep recommendations. Here, there is no difference between weekdays and weekends (35.9 % in each case). 

People in the low educational group are less likely to meet the recommendations than those in the high educational group (39.3 % vs. 62.5 %). The greatest differences between weekdays and weekends are seen in the medium educational group (46.5 % vs. 57.3 %). 

Overall, 33.1 % of adults sleep less than seven hours across the whole week, and 8.8 % sleep more than nine hours. In the 65 and older age group, 28.4 % sleep more than eight hours, 11.6 % more than nine hours, and 28.3 % less than seven hours. Among 18 – 29-year-olds, 26.8 % sleep less than seven hours and 12.0 % sleep more than nine hours. A detailed description of sleep duration by sleep category can be found in Table S1 of the Supplementary material. 

### Bedtimes 

Bedtimes differ significantly between weekdays and weekends (Figure 2). 

On weekdays, 38.1 % of adults go to bed after 22:00 and by 23:00, whilst at the weekend the prevalence is 32.1 % (Table 3). Late bedtimes (after 24:00) occur on weekdays among 11.5 % of men and 6.8 % of women; at weekends, the prevalences are 27.7 % and 15.8 % respectively. The proportion of 18–29-year-olds who go to bed late at weekends is particularly high (50.3 %), compared with 15.2 % on weekdays. Among people aged 65 and older, the proportion is significantly lower (8.8 % on weekdays and 10.0 % at the weekend). 8.2 % of people in the low educational group go to bed after 24:00 on weekdays and 13.0 % at the weekend. People in the high educational group are more likely to go to bed after 22:00 and by 23:00 on weekdays compared with those in the medium educational group (43.6 % vs. 37.2 %). At the weekend, the proportion of people who go to bed after 23:00 and by 24:00 is largest in the high educational group, at 34.7 %. 

### Relationship between sleep duration and bedtime 

Table 4 shows the probability of meeting the sleep recommendations, depending on bedtime. The predicted probability of meeting the sleep recommendations decreases clearly as bedtimes become later on weekdays. On weekdays, for women, it stands at 59.2 % for early bedtimes at or before 22:00 and falls to 25.5 % for late bedtimes after 24:00; a similar pattern is seen among men (52.0 % vs. 29.1 %). At the weekend, the probabilities are higher, particularly for moderate bedtimes after 22:00 and by 24:00 (60.0 % – 60.7 % for women and 52.3 % – 59.9 % for men) but decline slightly again for late bedtimes after 24:00 (women: 54.4 %; men: 49.8 %). 

## 4. Discussion

### Summary of the results

On average, adults in Germany sleep 07:26 hours per night, and around half (51.6 %) meet the recommended amount of sleep for their age group: seven to nine hours for those aged 18 – 64, and seven to eight hours for those aged 65 and older. As expected, sleep duration differs significantly among the adult population between weekdays and the weekend, and the recommendations are met more frequently at the weekend than on weekdays. There are also differences between population groups: women sleep significantly longer than men both on weekdays and at the weekend and are more likely to meet the recommended sleep duration. Young adults (aged 18 – 29) in particular sleep significantly longer at the weekend than on weekdays, whereas this difference is hardly observable among older adults. Overall, those aged 18 – 29 have the longest average sleep duration and those aged 45 – 64 the shortest. Adults aged 65 and older are the least likely to meet the recommendations on weekdays, at weekends, and over the whole week. Furthermore, a social gradient can be observed: adults in the higher educational group sleep longer on average and meet the recommendations more frequently than in the other groups. It should also be noted that the average sleep duration does not allow for a direct conclusion as to the proportion of people who fall within the recommended sleep duration range. Thus, there are hardly any differences in average sleep duration between educational groups, although differences in the prevalence of adherence to sleep recommendations are evident between these groups. Finally, the model analyses show that the highest probability of meeting the sleep recommendations on weekdays is associated with early bedtimes at or before 22:00 and, at weekends, after 22:00 and by 24:00. 

### Interpretation of the results

The differences in sleep duration between weekdays and weekends described here are also found in international population-based studies [31–33]. On weekdays, external demands such as working hours or family commitments often lead to shorter sleep duration, whilst at the weekend this can be compensated for by longer sleep durations [12, 13, 34]. This pattern is particularly pronounced among younger adults and decreases with older age, which is also observed in the results of this study and underlines the importance of occupational and social commitments as determinants of sleep duration [13]. 

The differences observed across different population groups are largely consistent with the existing literature. For instance, Saxvig et al. (2023) report an average sleep duration in a similar range within the Norwegian population, as well as shorter sleep durations among men compared to women [32]. These gender-specific differences are also supported by earlier studies: on average, women sleep longer than men [31, 35, 36] and, at the same time, exhibit higher sleep efficiency. This means that they spend a high proportion of their time in bed actually sleeping, even though they report sleep problems more frequently [37]. A study from the US has shown that this may, for example, be linked to the fact that whilst women sleep for longer periods, their sleep is more frequently interrupted by care work, which is proportionally more likely to be carried out by women [35]. Furthermore, hormonal fluctuations, such as those associated with the menstrual cycle, can affect the continuity and quality of sleep [37, 38]. There are currently no known gender-specific sleep recommendations. 

The age-related differences observed are also confirmed by other studies: longer sleep duration and a greater discrepancy in sleep duration between weekdays and weekends are found among young adults, whereas only minor differences are evident in older adults [31, 32]. The large discrepancy among young adults could be due to the fact that young adults tend to go to bed later than older adults. During the week, however, external constraints require them to get up earlier and consequently report shorter sleep durations. At the weekend, this sleep deficit is made up for, with significantly longer sleep durations being observed [32]. Furthermore, on days off, it is easier to follow one’s biological clock, and so-called ‘social jet lag’ can occur [31, 32]. This means that, due to the absence of external constraints, bedtimes on days off can be better adapted to the individual’s biological clock, resulting in significant differences in sleep times between weekdays and days off [18]. 

Studies also show that sleep duration is shortest between the ages of 40 and 50, which is consistent with the findings of the present analysis [38, 39]. On the one hand, people in this age group are usually engaged in intensive paid work with fixed working hours; on the other hand, there are social obligations, such as care work, which become more frequent in this age group [31, 32, 38]. In addition to these professional and social commitments, there are also biological changes. Both women and men may experience hormonal changes at this age, as well as changes in chronotypes [32, 38]. 

The low proportion of people aged 65 and older meeting the recommended sleep duration could be linked to the narrower recommended range for this age group: seven to eight hours, compared with seven to nine hours for younger adults. Consequently, people in this age group tend to sleep for longer than the recommended seven to eight hours. Furthermore, there may also be other factors that influence sleep duration in older people, such as physical or mental limitations [40].

The social differences observed are also consistent with the current state of research. Shorter sleep duration and more frequent sleep problems are found in groups with low socio-economic status and, in particular, in groups with low levels of education or low income [16, 41]. Possible explanations include less favourable working conditions, such as reduced flexibility in working hours or shift work, as well as a lower level of knowledge about sleep health. By contrast, people with a higher educational level are more likely to have working hours that can be better adapted to their individual sleep needs and, overall, exhibit more efficient sleep patterns [42–44]. 

### Strengths and limitations

The present analyses are based on data from a representative sample of the adult population in Germany. For the first time, detailed questions on sleep duration on weekdays and at weekends were asked, making it possible to quantify sleep duration across the entire adult population and to assess the extent to which the sleep duration recommendations are met. Furthermore, it is possible to differentiate according to socio-demographic characteristics. Previous studies on sleep behaviour have tended to cover relatively short survey periods of just a few weeks [22]. As the GEDA 2023 survey spanned several months, seasonal differences could be considered. However, as sleep behaviour in GEDA 2023 was not recorded over a full year, seasonal effects have not been examined in this article. Future studies should therefore enable year-round data collection to fully account for seasonal effects and reflect them accurately.

Further limitations arise from the study design and data collection methods. Questions about sleep times asked over the telephone may be inaccurate (self-reporting bias) and may also be distorted by memory (recall bias). The clustering of self-reported bedtimes around rounded times (response heaping, e.g. 22:00 or 22:30) leads to prevalence estimates being sensitive to the choice of cut-off points [45]. Objective measurement data, for example collected by wearables, could provide more valid data, but comes with higher costs. Comparisons of methods show that there are significant differences in the reports of sleep behaviour between objective and subjective data, and that sleep duration tends to be overestimated in self-reported data [46]. Furthermore, this survey did not collect data on activities prior to going to bed (e.g. reading, watching television, social media use). However, these could have an influence on the sleep duration and overall sleep health [47] and should be included in future surveys. Furthermore, the analysis only considered sleep periods at night and not during the day. It should also be noted that subjective sleep need does not necessarily correspond to the NSF’s population-based sleep recommendations. As subjective sleep need was not assessed in GEDA 2023, this aspect should be taken into account in future studies when considering potential adjustments to population-wide sleep recommendations. Analysing sleep behaviour across different occupational groups or in relation to shift work could also provide further insights into specific differences within the population [48, 49]. 

The results suggest that considering only the average sleep duration over the entire week may be potentially misleading. Whilst the average sleep duration falls within the recommended range, this is partly due to the fact that people sleep for less than seven hours on weekdays and tend to sleep for more than nine hours at the weekend, a pattern observed particularly among adults aged 18 to 29. Consequently, the recommendations are adhered to less frequently when weekdays and weekends are considered separately. This underlines the importance of a nuanced analysis of sleep patterns. In this context, there is already discussion as to the extent to which ‘catch-up sleep’ at the weekend may be beneficial to health [34]. However, irregular sleep schedules are also associated with a variety of adverse health outcomes, including metabolic, cardiovascular and mental health impairments [34]. It is therefore important to address the regularity of sleep schedules as well as adherence to the recommendations. 

Finally, it should be noted that simply considering the sleep duration does not allow any conclusions to be drawn about the sleep quality. To do so, further parameters, such as difficulties falling asleep and staying asleep, would need to be taken into account in the analyses [1, 50].

### Conclusion

This study presents, for the first time, a detailed analysis of sleep behaviour among the adult population in Germany, broken down by weekdays, weekends and the whole week. On average, only around half of the population achieves the recommended amount of sleep, with differences by gender, age group and educational group. The marked differences in sleep duration between weekdays and weekends, especially among younger adults, highlight the importance of social time structures, such as working hours, childcare and leisure activities. Sleep behaviour, as an important determinant of health, not only varies greatly from person to person but is also strongly influenced by external factors and social structures. Future research should therefore give greater consideration to the role of working conditions, care responsibilities and other societal factors in sleep health.

The findings also emphasise the need for a nuanced analysis of gender differences in sleep. In addition, the results suggest that bedtime plays an important role in meeting the recommendations: earlier bedtimes, particularly on weekdays, are associated with a higher likelihood of meeting the sleep duration recommendations. 

However, considering average sleep duration alone is not sufficient to comprehensively assess sleep behaviour and sleep health.

In order to comprehensively map sleep behaviour in future, the continuous recording of sleep parameters such as sleep quality and sleep disorders should be integrated into health monitoring to identify at-risk groups and target public health measures effectively.

## Figures and Tables

**Figure 1:**
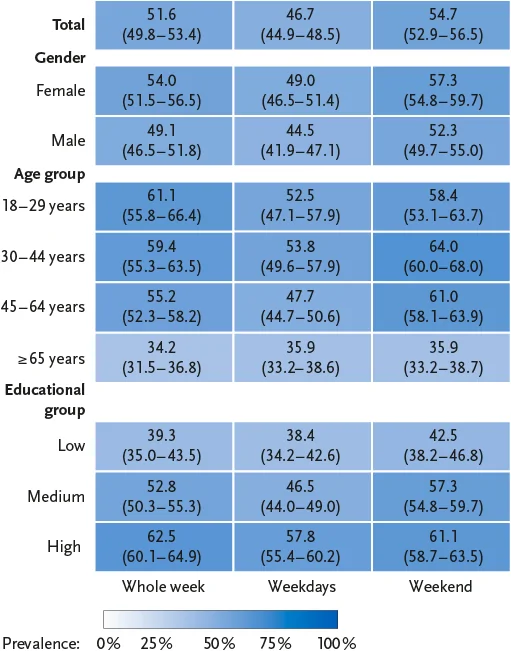
Weighted prevalences (%) of adherence to sleep recommendations by gender, age and educational groups for the whole week, weekdays and weekends (n = 3,878  women, n = 3,436 men). Source: GEDA 2023 Recommendation for adults aged 18 – 64: seven to nine hours Recommendation for adults aged 65 and older: seven to eight hours

**Figure 2:**
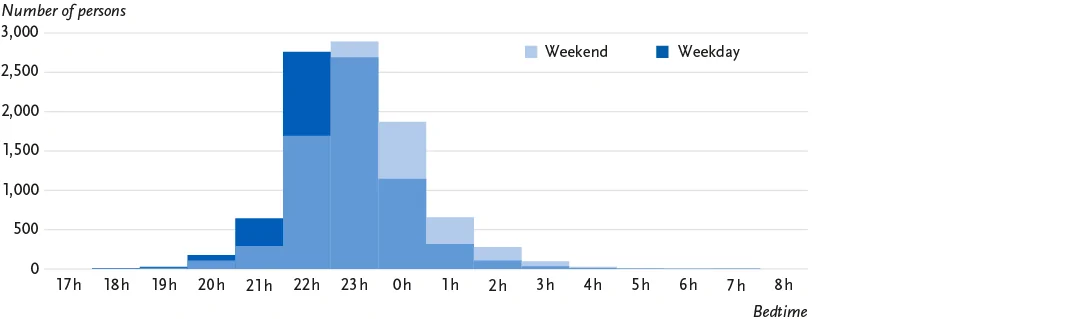
Distribution of bedtimes on weekdays and at weekends (n = 3,878 women, n = 3,436 men). Source: GEDA 2023

**Table 2:** Average sleep duration in hours:minutes per night on weekdays, at weekends and for the whole week, stratified by gender, age group and educational group (n = 3,878 women, n = 3,436 men). Source: GEDA 2023

	**Whole week**			**Weekdays**			**Weekend**		
	**Mean**	**(Median)**	**(95 % CI)**	**Mean**	**(Median)**	**(95 % CI)**	**Mean**	**(Median)**	**(95 % CI)**
**Total**	07:26	(07:29)	(07:23 – 07:28)	07:15	(07:20)	(07:12 – 07:18)	07:53	(07:55)	(07:50 – 07:56)
**Gender**									
Female	07:31	(07:34)	(07:27 – 07:35)	07:21	(07:25)	(07:17 – 07:25)	07:57	(08:00)	(07:53 – 08:01)
Male	07:20	(07:20)	(07:16 – 07:24)	07:08	(07:13)	(07:04 – 07:13)	07:49	(07:50)	(07:44 – 07:54)
**Age group**									
18 – 29 years	07:38	(07:42)	(07:30 – 07:45)	07:20	(07:23)	(07:11 – 07:29)	08:23	(08:25)	(08:14 – 08:31)
30 – 44 years	07:21	(07:27)	(07:15 – 07:28)	07:07	(07:15)	(07:00 – 07:15)	07:57	(07:58)	(07:50 – 08:04)
45 – 64 years	07:15	(07:17)	(07:11 – 07:19)	07:03	(07:00)	(06:58 – 07:08)	07:45	(07:50)	(07:40 – 07:50)
≥ 65 years	07:36	(07:42)	(07:31 – 07:41)	07:34	(07:40)	(07:29 – 07:38)	07:43	(07:50)	(07:38 – 07:48)
**Educational group**									
Low	07:28	(07:30)	(07:21 – 07:35)	07:18	(07:23)	(07:10 – 07:26)	07:54	(07:55)	(07:46 – 08:02)
Medium	07:23	(07:25)	(07:20 – 07:27)	07:12	(07:15)	(07:08 – 07:16)	07:53	(07:55)	(07:48 – 07:57)
High	07:29	(07:32)	(07:27 – 07:32)	07:20	(07:25)	(07:17 – 07:23)	07:54	(07:55)	(07:51 – 07:58)

95 % CI = 95 % confidence interval for the mean

**Table 1:** Characteristics of the entire study population stratified by gender, age and educational group (n = 3,878 women, n = 3,436 men). Source: GEDA 2023

	**n**	%^*^
**Total **	7,349	
**Gender**		
Female	3,878	51.5
Male	3,436	48.5
**Age group**		
18 – 29 years	631	16.1
30 – 44 years	1,135	22.8
45 – 64 years	2,662	34.8
≥ 65 years	2,921	26.2
**Educational group**		
Low	1,037	22.5
Medium	3,256	58.0
High	3,036	19.6
**Missing values:** Gender: n = 35; Educational group: n = 20		

^*^weighted

**Table 3:** Prevalence (%) of bedtimes on weekdays and at weekends, stratified by gender, age group and educational group (n = 3,878 women, n = 3,436 men). Source: GEDA 2023

**Weekdays**								
**Bedtime**	**≤ 22:00 h**		**> 22:00 – 23:00 h**		**> 23:00 – 24:00 h**		**> 24:00 h**	
	**%**	**(95 % CI)**	**%**	**(95 % CI)**	**%**	**(95 % CI)**	**%**	**(95 % CI)**
**Total**	33.6	(31.9 – 35.3)	38.1	(36.4 – 39.9)	19.2	(17.7 – 20.6)	9.1	(8.0 – 10.2)
**Gender**								
Female	36.5	(34.1 – 38.8)	40.2	(37.8 – 42.6)	16.6	(14.7 – 18.5)	6.8	(5.4 – 8.1)
Male	30.7	(28.3 – 33.2)	35.8	(33.4 – 38.3)	21.9	(19.7 – 24.0)	11.5	(9.7 – 13.3)
**Age group**								
18 – 29 years	23.1	(18.7 – 27.6)	36.8	(31.6 – 42.0)	24.9	(20.3 – 29.5)	15.2	(11.1 – 19.2)
30 – 44 years	37.0	(33.1 – 41.0)	36.4	(32.4 – 40.4)	17.9	(14.4 – 21.3)	8.7	(6.3 – 11.1)
45 – 64 years	40.1	(37.2 – 43.1)	38.0	(35.2 – 40.8)	15.0	(13.0 – 17.1)	6.8	(5.2 – 8.4)
≥ 65 years	28.3	(25.6 – 31.0)	40.6	(37.8 – 43.5)	22.3	(20.0 – 24.6)	8.8	(7.2 – 10.4)
**Educational group**								
Low	36.8	(32.6 – 40.9)	36.3	(32.3 – 40.3)	18.7	(15.4 – 22.1)	8.2	(5.7 – 10.7)
Medium	34.8	(32.4 – 37.2)	37.2	(34.8 – 39.6)	18.1	(16.2 – 20.1)	9.9	(8.3 – 11.5)
High	26.1	(23.8 – 28.4)	43.6	(41.1 – 46.1)	22.1	(20.0 – 24.2)	8.1	(6.9 – 9.4)
**Weekend**								
**Bedtime**	**≤ 22:00 h**		**> 22:00 – 23:00 h**		**> 23:00 – 24:00 h**		**> 24:00 h**	
	**%**	**(95 % CI)**	**%**	**(95 % CI)**	**%**	**(95 % CI)**	**%**	**(95 % CI)**
**Total**	16.9	(15.6 – 18.3)	32.1	(30.4 – 33.8)	29.4	(27.7 – 31.0)	21.6	(20.0 – 23.1)
**Gender**								
Female	19.6	(17.6 – 21.6)	36.2	(33.8 – 38.6)	28.5	(26.3 – 30.7)	15.8	(13.9 – 17.7)
Male	14.1	(12.3 – 16.0)	27.9	(25.6 – 30.2)	30.3	(27.9 – 32.7)	27.7	(25.2 – 30.1)
**Age group**								
18 – 29 years	7.5	(4.6 – 10.5)	15.8	(12.0 – 19.7)	26.4	(21.5 – 31.2)	50.3	(44.9 – 55.7)
30 – 44 years	14.9	(12.0 – 17.9)	35.6	(31.5 – 39.6)	28.6	(24.8 – 32.5)	20.9	(17.7 – 24.1)
45 – 64 years	18.9	(16.4 – 21.4)	31.2	(28.5 – 33.8)	32.5	(29.8 – 35.1)	17.5	(15.1 – 19.9)
≥ 65 years	21.9	(19.5 – 24.4)	40.3	(37.4 – 43.2)	27.8	(25.3 – 30.2)	10.0	(8.3 – 11.6)
**Educational group**								
Low	22.1	(18.6 – 25.6)	36.1	(32.1 – 40.0)	28.8	(24.8 – 32.8)	13.0	(10.1 – 16.0)
Medium	16.2	(14.4 – 18.1)	31.1	(28.8 – 33.4)	27.6	(25.4 – 29.8)	25.0	(22.8 – 27.3)
High	13.2	(11.4 – 15.0)	30.7	(28.5 – 33.0)	34.7	(32.3 – 37.1)	21.4	(19.3 – 23.5)

95 % CI = 95 % confidence interval

**Table 4:** Predicted probability (PP) of adhering to the recommended sleep duration depending on bedtime, stratified by gender (weighted) in % (n = 3,878 women, n = 3,436 men). Source: GEDA 2023

**Time**	**Women**		**Men**	
	**Weekdays**	**Weekend**	**Weekdays**	**Weekend**
	**PP (%)**	**PP (%)**	**PP (%)**	**PP (%)**
≤ 22:00 h	59.2	49.6	52.0	40.9
> 22:00 – 23:00 h	48.4	60.0	47.2	52.3
> 23:00 – 24:00 h	37.3	60.7	37.6	59.9
> 24:00 h	25.5	54.4	29.1	49.8
